# Supplementary material for: Causal Relationship between Aging and Anorexia Nervosa: A White-Matter-Microstructure-Mediated Mendelian Randomization Analysis
Source: Biomedicines. 2024 Aug 16;12(8):1874. doi: 10.3390/biomedicines12081874 (PMC11351342; doi:10.3390/biomedicines12081874)
Supplement: Supplementary file 1 [file biomedicines-12-01874-s001.zip › Supplementary Figure/Figure S4/Singe SNP plot of MR analysis for TL to ubm-b-2045.pdf]

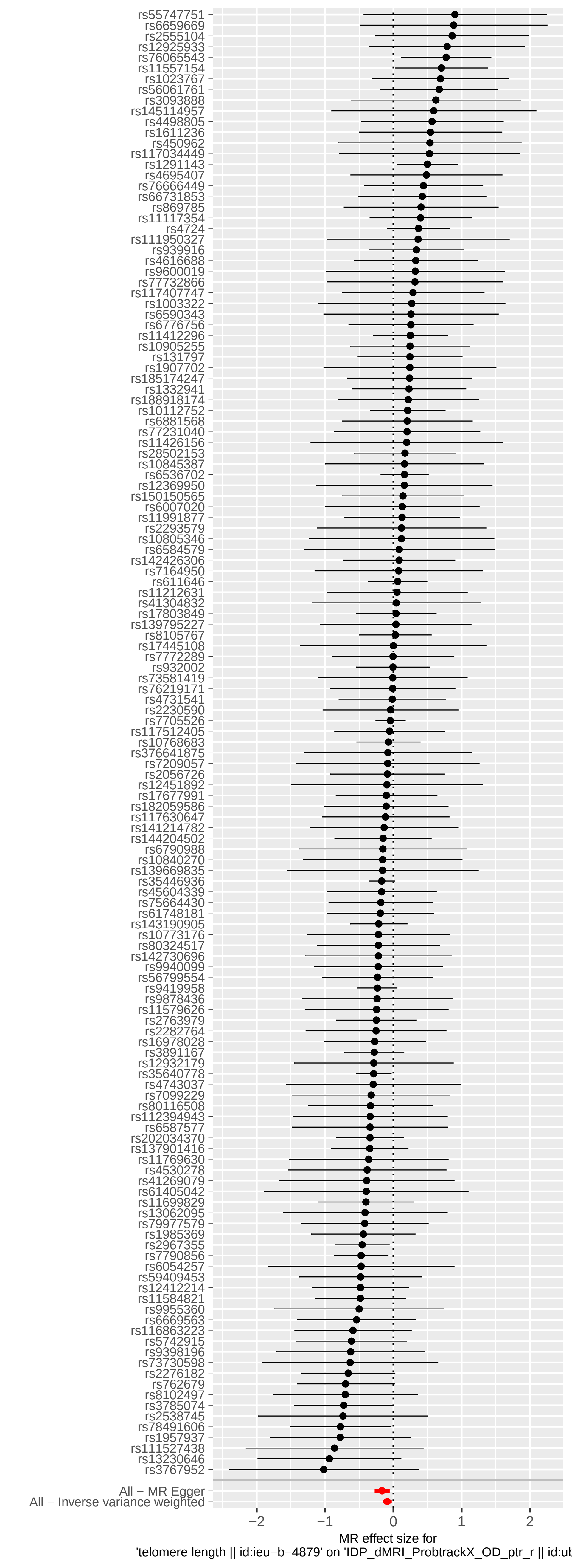
rs55747751
rs6659669
rs2555104
rs12925933
rs76065543
rs11557154
rs1023767
rs56061761
rs3093888
rs145114957
rs4498805
rs1611236
rs450962
rs117034449
rs1291143
rs4695407
rs76666449
rs66731853
rs869785
rs11117354
rs4724
rs111950327
rs939916
rs4616688
rs9600019
rs77732866
rs117407747
rs1003322
rs6590343
rs6776756
rs11412296
rs10905255
rs131797
rs1907702
rs185174247
rs1332941
rs188918174
rs10112752
rs6881568
rs77231040
rs11426156
rs28502153
rs10845387
rs6536702
rs12369950
rs150150565
rs6007020
rs11991877
rs2293579
rs10805346
rs6584579
rs142426306
rs7164950
rs611646
rs11212631
rs41304832
rs17803849
rs139795227
rs8105767
rs17445108
rs7772289
rs932002
rs73581419
rs76219171
rs4731541
rs2230590
rs7705526
rs117512405
rs10768683
rs376641875
rs7209057
rs2056726
rs12451892
rs17677991
rs182059586
rs117630647
rs141214782
rs144204502
rs6790988
rs10840270
rs139669835
rs35446936
rs45604339
rs75664430
rs61748181
rs143190905
rs10773176
rs80324517
rs142730696
rs9940099
rs56799554
rs9419958
rs9878436
rs11579626
rs2763979
rs2282764
rs16978028
rs3891167
rs12932179
rs35640778
rs4743037
rs7099229
rs80116508
rs112394943
rs6587577
rs202034370
rs137901416
rs11769630
rs4530278
rs41269079
rs61405042
rs11699829
rs13062095
rs79977579
rs1985369
rs2967355
rs7790856
rs6054257
rs59409453
rs12412214
rs11584821
rs9955360
rs6669563
rs116863223
rs5742915
rs9398196
rs73730598
rs2276182
rs762679
rs8102497
rs3785074
rs2538745
rs78491606
rs1957937
rs111527438
rs13230646
rs3767952
All − MR Egger
All − Inverse variance weighted
−2
−1
0
1
2
MR effect size for
'telomere length || id:ieu−b−4879' on 'IDP_dMRI_ProbtrackX_OD_ptr_r || id:ub
